# Serum-equivalency comparison, detection, and quantification of Group B *Streptococcus* anti-capsular polysaccharide antibodies from dried blood spots

**DOI:** 10.1080/21645515.2025.2544461

**Published:** 2025-08-14

**Authors:** Shanna Bolcen, Bailey Alston, Palak Y. Patel, Yikun Li, Panagiotis Maniatis, Donna Giordano Schmidt, Danka Pavliakova, Jessica E. Southwell, Lily Tao Jia, Michelle Gaylord, Raphael Simon, Natalie Clare Silmon de Monerri, Julia Rhodes, Stephanie Schrag, Sundaram Ajay Vishwanathan

**Affiliations:** a Division of Bacterial Diseases, National Center for Immunization and Respiratory Diseases, Centers for Disease Control and Prevention, Atlanta, GA, USA; b Eagle Global Scientific, Bristol Bay Native Corporation, Atlanta, GA, USA; c Seneca Federal Health, Seneca Nation Group Company, Chantilly, VA, USA; d IHRC, Inc, Atlanta, GA, USA; e Vaccine Research and Development, Pfizer Inc, Pearl River, NY, USA

**Keywords:** GBS, Group B *Streptococcus*, *Streptococcus agalactiae*, antibody, IgG, capsular polysaccharide, dried blood spot sample, serum, multiplex immunoassay

## Abstract

A standardized multiplex immunoassay (MIA) to quantify group B *Streptococcus* (GBS) anti-capsular polysaccharide (CPS) IgG serum concentrations was adopted by the Group B Streptococcal Assay Standardization (GASTON) consortium as a standardized serological assay with the most immediate applications for facilitating the licensure of GBS vaccines. However, dried blood spot (DBS) samples offer advantages for immunological studies, including cost-effectiveness, ease of transport, and storage. To determine suitability of DBS as an alternative sample matrix to serum in MIA, a contrived GBS seropositive panel, including matched DBS and serum samples, was prepared using established methods. The calculated geometric mean titers of GBS anti-CPS IgG values by individual serotype were compared using a paired t-test to establish serum equivalency. Geometric mean values for the matched panel were assessed via Deming regression for precision, accuracy, and concordance correlation coefficient (CCC). The initial acceptance criterion was set at 0.95 for CCC. Two additional criteria based on confidence intervals of CCC, slope, and intercept were used to determine the necessity of a serotype-specific conversion factor. The paired t-test *p*-values were >.05 for serum equivalency. For sample matrix concordance, CCC values were >0.95 and met correlation criteria for all serotypes. Conversion factors were applied to four serotypes (II, III, IV, and V) that did not meet the criteria for slope, intercept, or both. This demonstration of equivalency between DBS and serum supports the hypothesis that DBS is a suitable testing matrix from which to elucidate anti-CPS IgG concentrations in seroepidemiological and vaccine evaluation studies.

## Introduction

Group B *Streptococcus* (GBS), an encapsulated gram-positive pathogen, is a leading cause of neonatal and infant sepsis and meningitis in the first 90 days of life, contributing to infant morbidity and mortality globally. It has been associated with stillbirth, preterm delivery, and maternal invasive disease during pregnancy and in the post-partum period.^1,2^ Given the limitations of the currently recommended intrapartum antibiotic prophylaxis (IAP), development of standard of care maternal vaccines has been identified as a critical intervention to reduce the disease burden of neonatal GBS.^2–5^

High concentrations of serotype-specific maternal anti-capsular polysaccharide (CPS) IgG antibody are associated with protection and reduced infant disease via natural immunity.^6–9^ The CPS envelopes the bacterial cell and is a critical component in the ability to evade the host’s immune system defense mechanism of opsonophagocytosis and is therefore a key contributor to GBS’s virulence.^6^ Quantified anti-CPS IgG from case–control natural history studies may be used to determine antibody levels associated with protection via risk reduction curves and establish a correlate of protection for vaccine licensure.^2,7–9^ However, the use of variable assay methods and reagents in studies attempting to define anti-CPS IgG protective thresholds has complicated interpretation and limited progress toward globally accepted correlates of protection for neonatal invasive GBS disease.^2,9^

A Pfizer-developed 6-plex GBS CPS IgG direct immunoassay was adopted by the Group B Streptococcal Assay Standardization (GASTON) consortium as a standardized assay for quantification of IgG concentrations for the six predominant capsular serotypes of GBS: Ia, Ib, II, III, IV, and V.^9,10^ This GASTON multiplex immunoassay (MIA) was originally developed for serum, but alternative sample matrices will allow for greater flexibility in future studies. Dried blood spot (DBS) samples offer advantages over serum including overall reduced sample volume, cost, and need for special transport and storage conditions. In high-resource countries, DBS are routinely collected from neonates soon after birth as part of newborn screening programs to assess other health conditions. Remnant DBS samples from these collections offer an alternative to cord blood samples, which are not routinely stored for retrospective studies.^11–14^ DBS have proven to be a simple and powerful tool for large-scale investigation and seroepidemiological studies across multiple pathogens.^11–17^

In sampling from DBS, the choice of punch size (3 mm versus 6 mm) depends on specific test requirements and sample availability. The average volume for 3- and 6-mm punches is 1.6 ± 0.4 μL and 8.7 ± 1.9 μL, respectively.^11,18–20^ In this study (pre-print available^21^), IgG concentrations were measured to assess DBS punch size conditions for equivalency in addition to evaluating the correlation between serum and DBS anti-GBS CPS IgG concentrations, thus establishing DBS as a suitable alternative sample matrix for the standardized GASTON MIA.

## Materials and methods

### DBS and serum panel preparation

Thirty-one adult and two infant cord blood samples that spanned the dynamic range of the assay were prepared using fresh EDTA whole blood samples received from commercial biorepositories or through the Occupational Health and Wellness program at Pfizer (Pearl River, NY). Upon receipt, blood samples were centrifuged at 950 × g for 20 min. A calculated volume of the plasma fraction was replaced with an equivalent volume of sera from GBS6 immunized subjects (NCT03170609^22^) where all subjects underwent informed consent to obtain appropriate GBS anti-CPS IgG concentrations. Minimal volumes of the plasma fraction were replaced with GBS6 immunized sera to maintain EDTA concentrations to prevent coagulation. This contrived blood sample was then resuspended and used to prepare the DBS cards (PerkinElmer Health Sciences Inc., Catalog #GR2261002). DBS cards were prepared by adding 50 µL of the contrived blood sample onto the indicated spot on the DBS card, allowed to dry for ≥4 h, and then stored at −80°C with humidity sponges. The remaining contrived blood sample was then centrifuged at 950 × g for 20 min and the serum was removed and stored at −80°C until testing in the MIA.

### Elution of DBS

DBS were eluted using standardized practices^11^ with the MIA assay buffer serving as the elution buffer (0.5% BSA in 10 mM PBS/0.05% Tween-20/0.02% NaN_3_, pH 7.2). In brief, punches were taken from DBS cards using a manual, hand-held hole punch at the appropriate diameter and punched spots were collected directly into spin columns (0.45 µm Spin-X microcentrifuge filter tubes, Costar Catalog #8163). After adding 150 µL of elution buffer to the column, filter tubes were placed on an orbital shaker for 10 ± 5 min at room temperature (15°C to 30°C), after which the punched spots were checked for complete immersion and the tubes incubated overnight (16–20 h) at 2° to 8°C. After incubation, the orbital shaker procedure was repeated, and tubes were centrifuged at 15,000 × g for 3–5 min to collect the eluent. Total volume recovered was ≥90% of original elution buffer volume. This process is captured in Supplementary Figure S1.

### Multiplex immunoassay (MIA)

The previously published MIA^9,10^ was modified for application to DBS eluents while maintaining original assay sensitivity, limits of detection, and limits of quantification. In brief, for serum, samples diluted in assay buffer (0.5% BSA in 10 mM PBS/0.05% Tween-20/0.02% NaN3, pH 7.2) at 1:500, 1:5,000, and 1:50,000, quality control samples, and a human reference standard were added to a 96-well microtiter plate. Serotype-specific GBS CPS-poly-L-lysine conjugate coupled microspheres were added and then the plate incubated for 20 ± 4 h. After incubation, the plate was washed three times, and an R-Phycoerythrin-conjugated goat anti-human IgG secondary antibody (Jackson ImmunoResearch, 109–115–098) was added followed by a 90 ± 15-min incubation. The plate was then washed and read on a Bio-Plex 200 reader (Bio-Rad, Hercules, CA). For each sample, the signal output was expressed as serotype-specific median fluorescence intensity (MFI) and was then evaluated against the human reference standard for each serotype using a log–log linear regression. Serotype-specific anti-CPS IgG concentrations were reported out as µg/mL. In this study, elution of the DBS into 150 µL of buffer served as the initial dilution and dilution factor was determined by the number and size of DBS punches used (Table 1).

**Table 1.** Initial dilution and dilution series of dried blood spot samples by sample testing condition.

Sample	Initial Dilution	Dilution Series
One-3 mm punch	1:100	1:200, 1:2,000, 1:20,000
Two-3 mm punches	1:50	1:100, 1:1,000, 1:10,000
Three-3 mm punches	1:33.3	1:66.7, 1:667, 1:6,670
Four-3 mm punches	1:25	1:50, 1:500, 1:5000
One-6 mm punch	1:25	1:50, 1:500, 1:5000

### Punch size equivalency determination

Previous data equates a 3 mm punch to approximately 1.5 µL of serum.^11,18^ To verify expected serum-equivalent recovery rates between DBS punch sizes (3 mm and 6 mm), a panel of seven individual DBS samples was created. From each sample, one-, two-, three-, and four-3 mm (1/8 in) punches were compared against one-6 mm (1/4 in) punch. Each punch size condition was tested a minimum of three times on three different days by two independent operators resulting in a total ≥108 analytic endpoints.

### DBS to serum equivalency

To establish the correlation between serum and DBS using a serum reference standard, a panel of 33 matched DBS-serum sample sets as described above were tested for each serotype. Matched sample testing used the optimal number of 3 mm punches, established by the above-described punch size equivalency testing and was performed by two independent operators and repeated four times on four different days.

### Data and statistical analysis

Raw MFI values from the MIA were evaluated against a human serum reference standard curve with weight-based anti-CPS IgG assignments (µg/mL)^23^ using a log–log linear regression-based algorithm in a validated SAS application (Pfizer). This approach enables comparison of antibody concentrations across serotypes. All samples with a concentration coefficient of variation (CV) >30% were excluded from the analyses. Statistical analysis was modeled after previously published serum to DBS bridging experiments.^11^

Equivalence of log_10_-transformed sample anti-CPS IgG concentrations eluted from different punch sizes was assessed based on the coefficient of determination (R^2^) from regression analysis, with acceptance criteria set at ≥0.95. Additionally, the 95% confidence interval (CI) around the intercept needed to contain the value zero (indicating that at the baseline level, the two methods yield similar results with no significant systematic difference), and the 95% CI around the slope needed to contain the value 1 (indicating no significant proportional difference across the range). A paired t-test was used to assess individual serotypes once a target punch condition was selected for comparison against the 6 mm punch.

Equivalence between log_10_ transformed concentrations in paired serum and DBS eluents was assessed using the Concordance Correlation Coefficient (CCC), where the lower 95% confidence interval (CI) was set at ≥0.95.^11,24^ Deming regression^11,24^ between the two sample types was assessed to ascertain whether a serum-to-DBS conversion factor was needed. If the 95% CI for the intercept did not contain the value 0% or 95% CI for the slope did not contain the value 1, a conversion factor was applied.^25,26^

## Results

### Punch size equivalency determination

**Figure 1.**
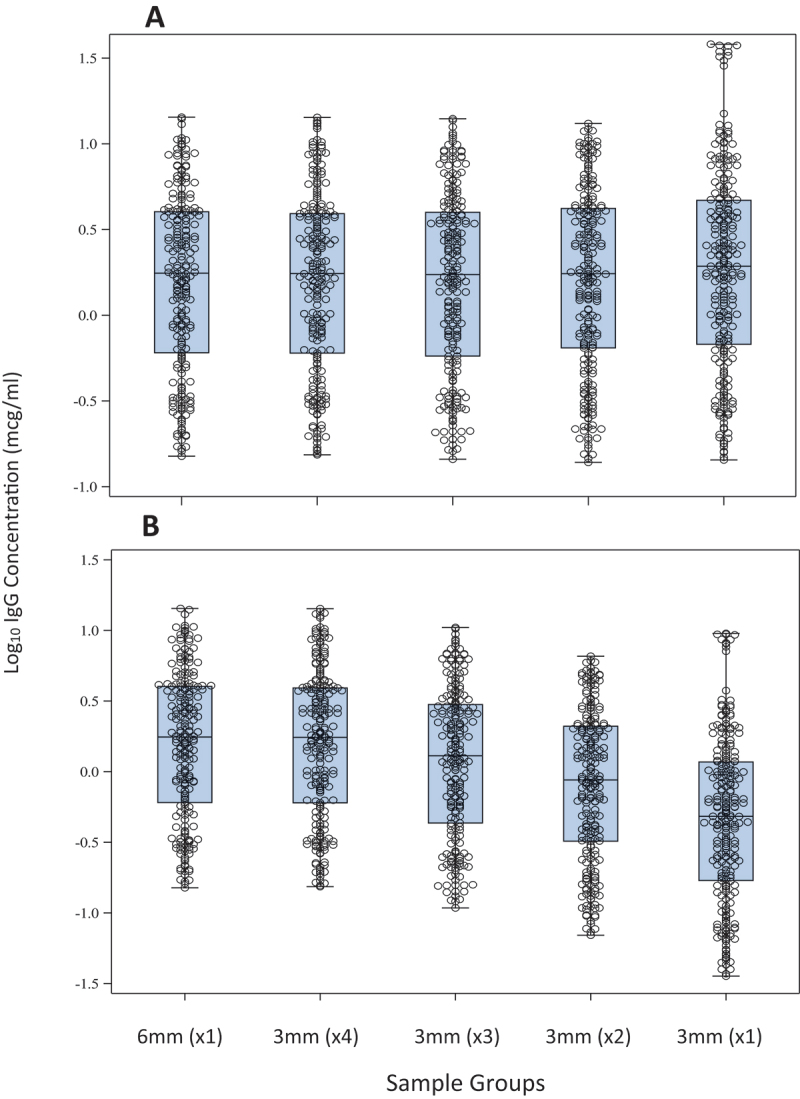
Box and scatter plot comparisons for the five sample punching conditions across all six serotypes with **A**. Final concentrations adjusted for individual sample dilution; **B**. Final concentrations held at the same dilution to assess the effect of the number of DBS punches.

**Figure 2.**
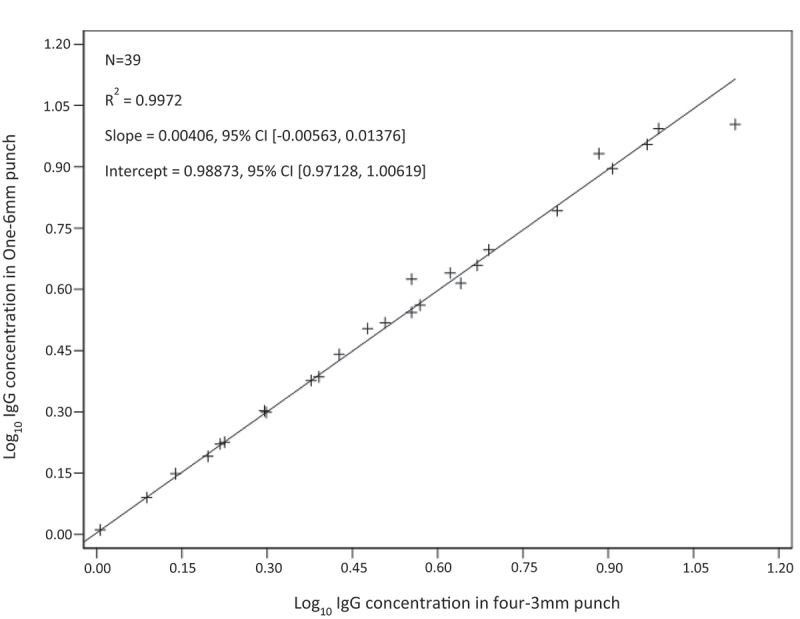
Linear regression with coefficient of determination (R^2^) and 95% CI of slope and intercept between sample IgG concentration eluted from four-3 mm DBS punch size and one-6 mm punch size.

The anti-CPS IgG geometric mean concentrations of the seven samples tested were compared by sample punch size condition against the one-6 mm punch anti-CPS IgG concentration (Figure 1). The four-3 mm punch condition was found to be equivalent to one-6 mm punch condition and was selected for additional evaluation. Anti-CPS IgG geometric mean concentrations across all six serotypes in the assay between the two punch size conditions were highly correlated (R^2^ = 0.9972) by regression analysis (Supplemental Table S1, Figure S2) and met the acceptance criteria set at >0.95 and 95% CI where the intercept contained the value 0 and slope contained the value 1 (Figure 2). Paired t-test *p*-values were >.05 [range 0.2031 (GBS Ib) −0.8317 (GBS IV)] for all serotypes in the four-3 mm punches versus one-6 mm punch comparison (Table 2).

**Table 2.** Paired t-test results for one-6 mm punch compared to 3 mm punches by serotype (pr (> |t|)) with final concentrations held at the same dilution.

Serotype	One-6 mm/four-3 mm (N=6)	One-6 mm/three-3 mm (N=6)	One-6 mm/two-3 mm (N=6)	One-6 mm/one-3 mm (N=6)
GBSIA	0.2724	0.0074	<.0001	<.0001
GBSIB	0.2031	<.0001	<.0001	<.0001
GBSII	0.7793	<.0001	<.0001	<.0001
GBSIII	0.5875	<.0001	<.0001	<.0001
GBSIV	0.8317	<.0001	<.0001	<.0001
GBSV	0.5524	0.0001	<.0001	<.0001

### DBS to serum equivalency

**Figure 3.**
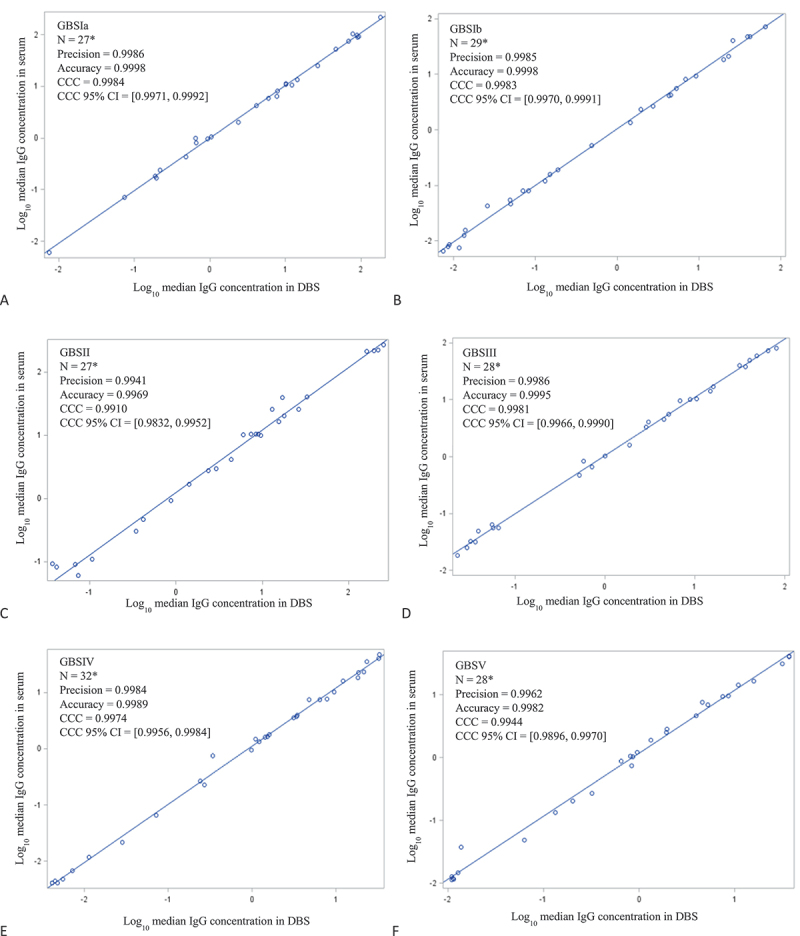
A–F. Deming regression of log-transformed serotype-specific anti-GBS IgG geometric mean concentrations determined in serum and DBS, showing CCC and other parameters.

Initial concordance between the DBS and serum tested was established by CCC by individual serotypes and all serotypes met the acceptable criteria with ranges from 0.9899 to 0.9981 (Figure 3). Accuracy and precision were ≥0.98 for all serotypes (Figure 3). The slope and intercept of the Deming regression best fit lines varied by serotype (Figure 3; Table 3). Criteria for conversion factor application (Table 3; Supplemental Table S2) for increased accuracy acceptance were met for two serotypes (GBS Ia and GBS Ib). For two serotypes (GBS II and GBS V), the 95% CI intercept did not contain the value 0 and, for one serotype (GBS III), the 95% CI for slope did not contain the value 1. Both intercept and slope criteria failed for GBS IV.

*Samples excluded from analysis due to sample CV. For detailed reportable values, refer to Supplementary Table S2.

**Table 3.** Summary of conversion formula for each serotype.

Serotype	Observed value with 95% Confidence interval (CI)		Conversion factor Applied (Yes/No)	Conversion formula
	Intercept	Slope		
GBSIa	−0.0022 [−0.0329, 0.0284]	1.0185 [0.9981, 1.0389]	No	NA
GBSIb	0.0189 [−0.0089, 0.0467]	1.0170 [0.9921, 1.0418]	No	NA
GBSII	0.0945 [0.0189, 0.1701]	0.9889 [0.9312, 1.0467]	Yes	LOG_VALUE = 0.0945 + log10(DBS_RESULT), CONVERTED_VALUE = 10**LOG_VALUE
GBSIII	0.0177 [−0.0098, 0.0453]	1.0251 [1.0044, 1.0459]	Yes	LOG_VALUE = 1.0251*log10(DBS_RESULT), CONVERTED_VALUE = 10**LOG_VALUE
GBSIV	0.0491 [0.0206, 0.0777]	1.0337 [1.0191, 1.0483]	Yes	LOG_VALUE = 0.0491 + 1.0337*log10(DBS_RESULT), CONVERTED_VALUE = 10**LOG_VALUE
GBSV	0.0703 [0.0317, 0.1090]	1.0045 [0.9637, 1.0454]	Yes	LOG_VALUE = 0.0703 + log10(DBS_RESULT), CONVERTED_VALUE = 10**LOG_VALUE

## Discussion

This manuscript presents a first study of its kind in a controlled setting to systematically demonstrate the suitability of DBS in the standardized multiplex immunoassay adopted by the GASTON consortium to quantify IgG antibodies against the most prevalent GBS CPS serotypes. The use of DBS has expanded to evaluate a plethora of biomarkers^27^ in recent years, given that collection is minimally invasive, requires less skilled labor, and uses fewer materials during sample collection compared to venipuncture. Additionally, DBS are easier and more efficient to transport from collection sites as they can be maintained at room temperature for up to 16 weeks without loss of biomarker functionality once the sample has dried and do not require cold-chain transportation.^11,13,14,17,27–29^ In addition to the utility of the sample matrix, DBS have been used in newborn screening programs since the 1960s and large sample collections are available for potential use in seroepidemiological studies.^27^ While sample stability is an important consideration when using DBS, assessing varying sample collection procedures and storage conditions fell outside the scope of this work and was not performed in this study.

Versatility in sample size collection from the DBS through different punch sizes allows for greater flexibility in testing when using remnant dried blood spot samples from historic collections. We successfully demonstrated punch size equivalency that verified previously established DBS-punch-size to serum-volume values.^18^ From the geometric mean anti-CPS IgG concentrations of the seven samples tested, a paired t-test was used to assess the significance of the differences between the arithmetic means of the various punch sizes of DBS. In this study, an alpha value of 0.05 (5%) was established as the criterion for significance. Using these criteria, it was determined that the four-3 mm punch size was the best fit for overall sample equivalency to the one-6 mm punch and could be used interchangeably in the assay. This allows the researcher to use 3 mm punches when larger punch sizes cannot be accommodated. These results thus add value by allowing the use of different punch sizes; for example, researchers could include specimens that have undergone prior testing such as remnant newborn screening samples, in which punches have already been removed from the filter paper.

In this study, the use of a 33-member matched serum and DBS panel allowed for the direct comparison of quantifiable anti-CPS IgG concentrations from two distinct sample matrices that were created from the same source material. The sample panel represented the range of the assay for the individual serotypes and ensured that the analysis captured the full spectrum of quantitative data obtained through the MIA. Despite the relatively small sample size, rigorous statistical methods were employed, including the concordance correlation coefficient (CCC) and strict criteria to analyze the data. These methods are designed to provide reliable estimates and confidence intervals, even with a limited sample set. In this assay, cross-laboratory reproducibility for the assay was found to be less than 25% relative standard deviation (RSD) for all six serotypes^9^ and the acceptable precision for the method is within 25% CV for results obtained within a single run.^10^ Therefore, study samples with a CV greater than 30% were excluded from analyses as they reflect excessive variability and don’t represent true assay performance.

When the matched DBS-serum sample pairs were tested, concordance >0.95 was demonstrated for all six GBS CPS serotypes captured by the assay. In addition to logarithmically transformed regression analysis, prior studies relied on CCC,^11^ Pearson correlation coefficient (PCC),^30^ Spearman’s rank correlation coefficient,^30^ or Cohen’s kappa coefficient^13,17^ to assess agreement between the sample matrices. For this study, CCC was selected to quantify the agreement, or concordance, between the two sample variables with acceptance set as the lower 95% CI > 0.95. This study also used an additional layer of stringency through the examination of the CI around the parameter estimates for the slope and intercept of the two sample groups. The use of a Deming regression accounted for errors in observations on both the x- and y-axis and provided a better estimate of the best line of fit for the data represented relationship between the DBS and serum. For this regression, the 95% CI must include 0, and slope must include 1. Finally, a DBS-to-serum conversion factor was applied to samples in which the slope or intercept failed to meet the above criteria.

Of the six GBS CPS serotypes assessed, four (GBS II, III, IV, and V) were found to increase result accuracy with the application of a constant multiplier as the conversion factor. While the application of a conversion factor introduces an additional layer of complexity in the practical application of this study’s finding, the use of conversion factors is a common practice in analytical methods when different sample matrices yield varying results and enhances accuracy and scientific rigor that outweigh the challenges. The conversion factors derived from Deming regression enable more precise comparisons between DBS and serum samples for the specific serotypes that did not meet the initial acceptance criteria and ensure that the results are not only comparable but also reflective of the true serological status of the samples. Future studies, using larger sample sizes, could provide more robust data and confirm the reliability of the conversion factors across different settings and could contribute to a better understanding of any underlying factors that contribute to the variability observed in specific serotypes.

In this study, bridging between the sample matrixes was performed on primarily adult specimens and may not be fully representative of infant samples that may have higher hematocrit concentrations. Hematocrit levels can affect the diffusion of the blood on the filter paper at the time of collection and influence the distribution of antibodies within the DBS sample.^31^ Ideally, to overcome this potential hematocrit effect, the entire DBS sample would be utilized in testing to ensure that the variation of diffusion is adequately captured. However, this study utilized remnant newborn screening samples with punches removed for prior testing. To mitigate the hematocrit effect and given sample limitations, this study utilized random sampling of the DBS when taking the necessary four 3-mm punches to offset any difference in diffusion rates.

In a controlled setting using contrived panels, the use of DBS as a testing matrix in the quantification of GBS anti-CPS antibodies was successfully demonstrated. These results not only advance GBS-related seroepidemiological studies with DBS but also impact the work of the GASTON consortium and the broader GBS community striving for licensure of a maternal vaccine against invasive GBS disease in infants.

## Supplementary Material

Serum_equiv_Comp_Detect_Quant_of_GBS_Abs_Supp_RevisedClean.docx

## Data Availability

The data supporting the findings of this study are available within the article and its supplementary materials.
