# Supplementary material for: Serum-equivalency comparison, detection, and quantification of Group B Streptococcus anti-capsular polysaccharide antibodies from dried blood spots
Source: Hum Vaccin Immunother. 2025 Aug 14;21(1):2544461. doi: 10.1080/21645515.2025.2544461 (PMC12355705; doi:10.1080/21645515.2025.2544461)
Supplement: Serum_equiv_Comp_Detect_Quant_of_GBS_Abs_Supp_RevisedClean.docx [file KHVI_A_2544461_SM6257.docx]

**Title:** Serum-equivalency Comparison, Detection, and Quantification of Group B *Streptococcus* Anti-capsular Polysaccharide Antibodies from Dried Blood Spots

**Author names:** Shanna Bolcen^1^, Bailey Alston^2,4^, Palak Y. Patel^1,*^, Yikun Li^3,*^, Panagiotis Maniatis^1^, Donna Giordano Schmidt^4^, Danka Pavliakova^4^, Jessica E. Southwell^2,3^, Lily Tao Jia^1^, Michelle Gaylord^4^, Raphael Simon^4^, Natalie Clare Silmon de Monerri^4^, Julia Rhodes^1^, Stephanie Schrag^1^, Sundaram Ajay Vishwanathan^1^

**Authors’ affiliations:**

^1^ Division of Bacterial Diseases, National Center for Immunization and Respiratory Diseases, Centers for Disease Control and Prevention, 1600 Clifton Rd NE, Atlanta, GA 30329

^2^ Eagle Global Scientific, 2835 Brandywine Rd, Suite 200, Atlanta, GA 30341

^3^ IHRC, Inc, 2 Ravinia Dr, Suite 1200, Atlanta, GA 30346

^4^ Seneca Federal Health, 14200 Park Meadow Dr, Suite 125-N, Chantilly, VA 20151

^5^ Vaccine Research and Development, Pfizer Inc. Pearl River, New York, 401 North Middletown Road, Pearl River, New York 10965

* Denotes equal contribution

Address correspondence to Shanna Bolcen ([fky0@cdc.gov](mailto:fky0@cdc.gov)); ORCID 0000-0002-7464-9967; Division of Bacterial Diseases, MPDLB, CDC, Atlanta, GA 30329, USA. 404 718 1490 (Tel)

# Supplementary Information

**Supplementary Figure 1.** Elution process for dried blood spots samples in the multiplex immunoassay test flow.

**Supplementary Table 1.** Reportable values (mcg/mL) used for punch size analysis.

| **Samples (N=42)** | **Serotype** | **3 mm(x1)** | **3 mm(x2)** | **3 mm(x3)** | **3 mm(x4)** | **6 mm(x1)** |
| --- | --- | --- | --- | --- | --- | --- |
| PFDBS1 | GBSIA | 0.89871 | 1.93840 | 2.82525 | 3.7055 | 3.6406 |
| PFDBS1 | GBSIB | 0.32522 | 0.68144 | 0.94936 | 1.2245 | 1.2311 |
| PFDBS1 | GBSII | 1.41544 | 2.64031 | 4.01959 | 4.8953 | 4.9719 |
| PFDBS1 | GBSIII | 0.62046 | 1.27452 | 1.86104 | 2.3833 | 2.3808 |
| PFDBS1 | GBSIV | 0.69051 | 1.48388 | 2.08008 | 2.4594 | 2.4309 |
| PFDBS1 | GBSV | 0.40859 | 0.88127 | 1.29058 | 1.6783 | 1.6823 |
| PFDBS2 | GBSIA | 0.36904 | 0.78804 | 1.03159 | 1.5695 | 1.5520 |
| PFDBS2 | GBSIB | 0.07384 | 0.16125 | 0.24179 | 0.3270 | 0.3227 |
| PFDBS2 | GBSII | 0.44295 | 1.01548 | 1.52927 | 1.9872 | 1.9892 |
| PFDBS2 | GBSIII | 0.15095 | 0.30806 | 0.46549 | 0.6451 | 0.6400 |
| PFDBS2 | GBSIV | 0.19398 | 0.42919 | 0.68127 | 0.9289 | 0.9118 |
| PFDBS2 | GBSV | 0.10769 | 0.20960 | 0.28115 | 0.4367 | 0.4136 |
| PFDBS3 | GBSIA | 8.21544 | NA* | NA* | NA* | NA* |
| PFDBS3 | GBSIB | 0.04044 | 0.08125 | 0.12636 | 0.1746 | 0.1785 |
| PFDBS3 | GBSII | 0.24230 | 0.46353 | 0.69341 | 1.0144 | 1.0256 |
| PFDBS3 | GBSIII | 0.07107 | 0.14647 | 0.22814 | 0.3338 | 0.3303 |
| PFDBS3 | GBSIV | 0.07911 | 0.15078 | 0.22745 | 0.3214 | 0.3043 |
| PFDBS3 | GBSV | 0.05094 | 0.10750 | 0.15279 | 0.2135 | 0.2170 |
| PFDBS4 | GBSIA | 9.06573 | NA* | NA* | NA* | NA* |
| PFDBS4 | GBSIB | 0.60771 | 1.27564 | 1.89106 | 2.6713 | 2.7587 |
| PFDBS4 | GBSII | 2.22713 | 4.43688 | 6.05359 | 7.6489 | 8.5545 |
| PFDBS4 | GBSIII | 0.97486 | 2.06428 | 3.00357 | 4.1861 | 4.3634 |
| PFDBS4 | GBSIV | 1.10533 | 2.26648 | 3.17831 | 4.6740 | 4.5565 |
| PFDBS4 | GBSV | 0.71333 | 1.50579 | 2.18975 | 2.9971 | 3.1910 |
| PFDBS6 | GBSIA | 2.65197 | 5.10661 | 8.07837 | 9.7404 | 9.8556 |
| PFDBS6 | GBSIB | 0.05807 | 0.11107 | 0.16930 | 0.2192 | 0.2293 |
| PFDBS6 | GBSII | 0.45947 | 0.84312 | 1.38199 | 1.9730 | 2.0075 |
| PFDBS6 | GBSIII | 0.09232 | 0.17201 | 0.27530 | 0.3514 | 0.3738 |
| PFDBS6 | GBSIV | 1.11250 | 2.00531 | 2.88578 | 3.5811 | 4.2166 |
| PFDBS6 | GBSV | 0.14533 | 0.27156 | 0.40487 | 0.5229 | 0.5637 |
| PFDBS7 | GBSIA | 1.94490 | 4.03727 | 5.62684 | 8.0797 | 7.8554 |
| PFDBS7 | GBSIB | 0.86673 | 1.77934 | 2.50030 | 3.5814 | 3.4950 |
| PFDBS7 | GBSII | 3.18553 | 5.50562 | 8.02097 | 13.2861 | 10.0855 |
| PFDBS7 | GBSIII | 1.62619 | 3.34439 | 4.55858 | 6.4612 | 6.2022 |
| PFDBS7 | GBSIV | 2.17933 | 4.53192 | 6.59672 | 9.3024 | 9.0075 |
| PFDBS7 | GBSV | 1.24500 | 2.59004 | 3.63882 | 4.3704 | 4.1195 |
| PFDBS8 | GBSIA | NA* | NA* | NA* | NA* | NA* |
| PFDBS8 | GBSIB | 0.18524 | 0.38052 | 0.61543 | 0.8013 | 0.8250 |
| PFDBS8 | GBSII | 0.65815 | 1.39101 | 2.37723 | 3.2175 | 3.2980 |
| PFDBS8 | GBSIII | 0.38675 | 0.77788 | 1.18764 | 1.6489 | 1.6642 |
| PFDBS8 | GBSIV | 0.29682 | 0.54462 | 1.00651 | 1.3769 | 1.4078 |
| PFDBS8 | GBSV | 0.21364 | 0.41424 | 0.64737 | 0.8949 | 0.9377 |

* NA; Data not available (no valid result).

| **Supplementary Table 2.** Reportable values (concentration mcg/mL) used for DBS-Serum bridging analysis. | | | | | | | | | | | | | |
| --- | --- | --- | --- | --- | --- | --- | --- | --- | --- | --- | --- | --- | --- |
| **DBS Sample (N=33)** | **Serum Sample (N=33)** | **Anti-GBSIa IgG** | | **Anti-GBSIb IgG** | | **Anti-GBSII IgG** | | **Anti-GBSIII IgG** | | **Ant-GBSIV IgG** | | **Anti-GBSV IgG** | |
|  |  | **DBS** | **Serum** | **DBS** | **Serum** | **DBS** | **Serum** | **DBS** | **Serum** | **DBS** | **Serum** | **DBS** | **Serum** |
| **PFDBC3-500** | **PFCRS3** | **0.221** | **0.242** | **0.151** | **0.157** | **197.27** | **216.931** | **77.672** | **NA**** | **0.269** | **0.23** | **0.133** | **0.135** |
| **PFDBC4-500** | **PFCRS4** | **76.981** | **NA**** | **0.014** | **0.016** | **NA*** | **184.657** | **31.309** | **40.213** | **1.106** | **1.469** | **0.022** | **NA*** |
| **PFDA01-500** | **PFSE01** | **93.171** | **92.048** | **0.485** | **0.516** | **439.006** | **NA**** | **65.057** | **72.289** | **32.627** | **40.62** | **37.187** | **40.222** |
| **PFDA02-500** | **PFSE02** | **255.19** | **NA**** | **0.187** | **0.192** | **NA*** | **148.651** | **40.876** | **49.204** | **1.439** | **1.614** | **7.524** | **9.309** |
| **PFDA03-500** | **PFSE03** | **4.136** | **4.28** | **1.454** | **1.335** | **552.8** | **NA**** | **36.854** | **37.629** | **7.795** | **7.652** | **31.408** | **30.878** |
| **PFDA04-500** | **PFSE04** | **152.254** | **NA**** | **1.949** | **2.319** | **161.616** | **209.681** | **48.621** | **59.868** | **12.22** | **15.812** | **0.063** | **0.048** |
| **PFDA05-500** | **PFSE05** | **69.03** | **75.498** | **65.396** | **71.576** | **NA**** | **NA**** | **NA*** | **NA*** | **0.24** | **0.268** | **0.644** | **0.865** |
| **PFDA06-500** | **PFSE06** | **88.507** | **96.896** | **6.868** | **8.208** | **7.425** | **10.313** | **0.576** | **0.829** | **4.787** | **7.396** | **0.014** | **0.037** |
| **PFDA07-500** | **PFSE07** | **209.129** | **NA**** | **39.343** | **47.979** | **2.358** | **2.76** | **0.055** | **0.063** | **1.222** | **1.318** | **1.334** | **1.885** |
| **PFDA08-500** | **PFSE08** | **77.815** | **102.846** | **26.12** | **40.262** | **6.027** | **10.044** | **6.795** | **9.65** | **0.341** | **0.747** | **4.592** | **7.59** |
| **PFDA10-500** | **PFSE10** | **0.648** | **0.987** | **0.026** | **0.043** | **NA**** | **NA**** | **3.035** | **4.099** | **23.245** | **35.141** | **NA**** | **NA**** |
| **PFDA12-500** | **PFSE12** | **181.544** | **218.194** | **42.367** | **47.982** | **254.207** | **269.112** | **NA**** | **NA**** | **32.909** | **47.09** | **79.924** | **NA**** |
| **PFDA13-500** | **PFSE13** | **0.937** | **0.951** | **0.07** | **0.081** | **13.099** | **25.72** | **2.881** | **3.279** | **6.414** | **7.485** | **0.81** | **1.046** |
| **PFDA16-500** | **PFSE16** | **10.144** | **11.045** | **5.315** | **5.623** | **33.047** | **39.909** | **8.826** | **10.166** | **18.435** | **22.149** | **0.951** | **1.222** |
| **PFDA17-500** | **PFSE17** | **46.502** | **52.627** | **0.049** | **0.055** | **17.869** | **20.43** | **5.095** | **5.497** | **3.459** | **3.908** | **10.905** | **14.263** |
| **PFDA18-500** | **PFSE18** | **14.519** | **13.551** | **22.881** | **21.135** | **9.575** | **9.903** | **0.039** | **0.049** | **18.294** | **18.112** | **37.165** | **41.241** |
| **PFDA19-500** | **PFSE19** | **90.805** | **89.864** | **4.505** | **4.289** | **219.748** | **224.495** | **14.9** | **14.298** | **0.988** | **0.954** | **3.98** | **4.596** |
| **PFDA20-500** | **PFSE20** | **26.757** | **25.078** | **4.336** | **4.085** | **26.57** | **25.525** | **80.831** | **81.965** | **3.133** | **3.572** | **8.653** | **9.503** |
| **PFDA21-500** | **PFSE21** | **7.87** | **8.224** | **20.052** | **18.591** | **1.424** | **1.675** | **10.565** | **10.434** | **1.536** | **1.629** | **1.936** | **2.502** |
| **PFDA22-500** | **PFSE22** | **0.653** | **0.8** | **0.012** | **0.007** | **17.139** | **39.297** | **16.057** | **17.107** | **21.434** | **23.305** | **1.955** | **2.835** |
| **PFDA23-500** | **PFSE23** | **1.046** | **1.052** | **9.229** | **9.299** | **8.428** | **10.396** | **0.057** | **0.055** | **3.379** | **3.727** | **15.829** | **16.503** |
| **PFDA24-500** | **PFSE24** | **5.944** | **5.868** | **2.721** | **2.682** | **15.551** | **16.348** | **4.514** | **4.536** | **9.434** | **10.194** | **0.854** | **1.035** |
| **PFDA25-500** | **PFSE25** | **10.118** | **11.201** | **0.082** | **0.08** | **9.035** | **10.319** | **1.001** | **1.036** | **1.607** | **1.784** | **5.218** | **6.88** |
| **PFDA26** | **PFSE26** | **0.189** | **0.183** | **0.009** | **0.009** | **0.883** | **0.931** | **0.719** | **0.653** | **0.004** | **0.004** | **0.013** | **0.015** |
| **PFDA28** | **PFSE28** | **0.007** | **0.006** | **0.007** | **0.006** | **0.346** | **0.301** | **NA**** | **NA**** | **0.004** | **0.004** | **0.322** | **0.271** |
| **PFDA31** | **PFSE31** | **2.395** | **2.046** | **NA**** | **NA**** | **0.041** | **0.083** | **0.023** | **0.018** | **0.028** | **0.022** | **0.831** | **0.74** |
| **PFDA33** | **PFSE33** | **7.726** | **6.49** | **0.05** | **0.047** | **0.037** | **0.094** | **NA**** | **NA**** | **0.011** | **0.012** | **NA**** | **NA**** |
| **PFDA35** | **PFSE35** | **0.479** | **0.428** | **NA**** | **NA**** | **0.074** | **0.06** | **0.036** | **0.031** | **0.071** | **0.066** | **0.201** | **0.201** |
| **PFDA37** | **PFSE37** | **12.106** | **10.586** | **0.131** | **0.12** | **0.068** | **0.09** | **0.065** | **0.056** | **0.007** | **0.007** | **0.011** | **0.013** |
| **PFDA39** | **PFSE39** | **NA*** | **0.002** | **NA**** | **NA**** | **4.394** | **4.136** | **0.523** | **0.467** | **0.005** | **0.004** | **NA**** | **NA**** |
| **PFDA41** | **PFSE41** | **0.066** | **NA*** | **0.013** | **0.013** | **2.915** | **2.941** | **1.885** | **1.609** | **0.008** | **NA*** | **0.011** | **0.011** |
| **PFDA42** | **PFSE42** | **0.197** | **0.167** | **0.009** | **0.008** | **0.422** | **0.459** | **0.029** | **0.025** | **0.005** | **0.005** | **0.011** | **0.012** |
| **PFDA43** | **PFSE43** | **0.075** | **0.071** | **NA**** | **NA**** | **0.107** | **0.11** | **0.032** | **0.033** | **0.004** | **0.004** | **0.011** | **0.011** |
| **Paired Data used in Deming Regression analysis (N)** | | **N=27** | | **N=29** | | **N=27** | | **N=28** | | **N=32** | | **N=28** | |

NA*: Data not available (sample results with CV > 30%)

NA**: Data not available (non-detectable result).
